# Saturated fatty acid palmitate-induced insulin resistance is accompanied with myotube loss and the impaired expression of health benefit myokine genes in C2C12 myotubes

**DOI:** 10.1186/1476-511X-12-104

**Published:** 2013-07-18

**Authors:** Ming Yang, Dandan Wei, Chunfen Mo, Jie Zhang, Xu Wang, Xiaojuan Han, Zhe Wang, Hengyi Xiao

**Affiliations:** 1 Department of Geriatrics, Regenerative Medicine Research Center, Laboratory of Stem cell biology, State Key Laboratory of Biotherapy, West China Hospital, Sichuan University, Keyuan 4-1, Gaopeng Street, High-tech Zone, Chengdu 610041, China

**Keywords:** Palmitate, Myotube loss, Myokine, Insulin resistance

## Abstract

**Background:**

Excessive circular fatty acid, particlarly saturated fatty acid, can result in insulin resistance in skeletal muscle, but other adverse effects of fatty acid accumulation in myocytes remain unclear.

**Methods:**

Differentiated C2C12 myotubes were used. The effects of palmitate on cell viability, glucose uptake, gene expression and myotube loss were evaluated by MTT assay, 2NBDG uptake, qRT-PCR, Western Blot and crystal staining-based myotube counting, respectively. In some expreiments, oleate was administrated, or the inhibitors of signaling pathways were applied.

**Results:**

Palmitate-induced cellular insulin resistance was clarified by the reduced Akt phosphorylation, glucose uptake and Glut4 expression. Palmitate-caused myotube loss was clearly observed under microscope and proved by myotube counting and expression analysis of myotube marker genes. Moreover, palmitate-induced transcriptional suppression of three health benefit myokine genes (FNDC5, CTRP15 and FGF21) was found, and the different involvement of p38 and PI3K in the transcription of these genes was noticed.

**Conclusions:**

Palmitate-induced insulin resistance accompanys myotube loss and the impaired expression of FNDC5, CTRP15 and FGF21genes in C2C12 myotubes. These results provide novel evidence indicating the negative role of high concentration of palmitate in myotubes.

## Background

Insulin resistance is one of the major characteristics of type 2 diabetes mellitus (T2D) and also occurs with obesity, hypertension and cardiovascular disease [1,2]. Excessive high level plasma free fatty acids (FFA) is known to associate with insulin resistance in diabetic patients and nondiabetic subjects [3,4]. Correspondingly, an impairment of glucose use and insulin sensitivity has been observed in experimental studies with high concentration FFA administration. As skeletal muscle accounts for more than 70% of insulin-stimulated glucose consumption, its status can obviously affect whole body plasma glucose concentration and insulin sensitivity [2]. In fact, high concentration of FFA, particularly the most abundant dietary saturated fatty acid palmitate, can directly impair insulin signaling in skeletal muscle cells [5,6].

The mechanism underlying palmitate-induced insulin resistance remains unclear, with one hypothesis indicating that palmitate acts through protein kinase C (PKC) to negatively regulate the ability of IRS-1 to activate PI3K-Akt pathway [7,8]. Meanwhile, our understanding about the consequences of palmitate-induced insulin resistance in myotubes is limited. The most known one is decreased glucose uptake [9,10].

Myotube formation is a morphologic and functional feature of skeletal muscle. It shapes upon a homeostasis between myofiber synthesis and proteolysis [11,12]. As known, myosin heavy chain (MHC) proteins are important myofiber components and muscle creatine kinase (MCK) is a muscle specific ATPase necessary for myofiber assembly and contraction [11]. Moreover, A study demonstrates that palmitate has negative effect on the myotube size and morphology in differentiated C2C12 cells [13]. Moreover, it has found that myotube atrophy or myotube loss is a common syndrome of late T2D and other catabolic diseases [14-16]. But, if and how the high level of fatty acids affects myotube homeostasis is still an open question.

Diverse myokines are produced by muscle cells [17]. Some of them, such as irisin (N-terminal portion of FNDC5 pro-protein), CTRP15 and fibroblast growth factor-21 (FGF21), have attracted an increasing attention in recent years because of their potential beneficial roles in metabolic homeostasis and protecting human body from the damages of metabolic diseases [18-20]. However, little is known about the connection between high fatty acids and the expression of myokine genes in C2C12 myotubes.

The purpose of this study, therefore, is to investigate the influence of palmitate in muscle fiber composition and the expression of FNDC5, CTRP15 and FGF21 genes. The signaling pathways involved are also preliminarily investigated.

## Materials and methods

### Materials

2-(N-(7-nitrobenz-2-oxa-1,3-diazol-4-yl)-amino)-2-deoxyglucose (2NBDG) was from Invitrogen, palmitate from Sigma, oleate from Alligator Reagent, fatty free BSA from MP Biomedicals, LY294002 and SB203580 and MG132 from Calbiochem.

### Cell culture and differentiation

Mouse C2C12 myoblasts (American Type Culture Collection) were maintained in Dulbecco’s modified Eagle’s medium (DMEM) supplemented with 10% FBS. C2C12 myotubes were obtained by culturing myoblasts in DMEM containing 2% heat-inactivated horse serum for at least 4 days.

### Fatty acids preparation and cell treatment

Palmitate was prepared as described previously [21]. Briefly, palmitate was dissolved in 0.1M NaOH by heating at 70°C. After filtration, the solution was then diluted with 10% fatty free BSA and stored at −20°C. Oleate was dissolved in PBS by heating at 55°C, and diluted with 10% fatty free BSA and stored at −20°C. Palmitate and oleate treatments were performed with the concentrations indicated in figure legends for generally 24 hours as described previously [22]. In all experiments, 2 hours before the treatment of fatty acids, cultural medium were changed to serum free DMEM. For protein phosphorylation detection, 100 nM insulin was added for 15 min before cell lysates harvest. In some experiments, cells were pretreated with LY294002, SB203580, MG132 or vehicle for 1 hour before stimulating with palmitate.

### Cell viability assay

Cell viability was measured using the MTT (3-(4, 5)-dimethylthiahiazo (−z-y1)-3, 5-diphenytetrazoliumromid) assay, based on the MTT conversion into formazan crystals using mitochondrial dehydrogenases. Briefly, C2C12 cells were plated at a density of 2×10^4^ cells/well in a 96-well plate. After differentiation and palmitate treatment for 24 hours, 15 μl of 5 mg/ml MTT was added to each well. After 4 hours incubation at 37°C, this solution was removed carefully and the produced formazan was solubilized in 150 μl dimethyl sulfoxide (DMSO). The absorbance was measured at 490 nm using a microplate reader (Bio-Rad).

### Measurement of 2NBDG uptake

After 2 hours incubation in no glucose DMEM, myotubes were incubated with or without 100 nM insulin for another 1 hour. Next, myotubes were transferred to fresh no glucose DMEM medium supplemented with 80 uM fluorescent deoxyglucose 2NBDG for 30 min. After three times washed by PBS, myotubes were lysed by 0.5% TritonX-100 and the fluorescence intensity was recorded using a microplate reader (Thermo) at excitation and emission wavelengths of 485 and 538 nm, respectively.

### Crystal violet staining

Cells were fixed for 10 min with 4% paraformaldehyde (PFA), then stained with 1% crystal violet for 5min, and washed two times with water.

### Myotube counting

After crystal violet staining, nine visual fields for each treatment were photographed under a microscope. The number of myotubes in these photographs was determined following counting. Myotubes were identified as obviously bigger or longer morphology than undifferentiated C2C12 myoblasts.

### Real time-PCR

Total RNA extraction, reverse transcription reaction and quantitative real-time PCR assays were performed as described previously [23]. Briefly, total RNA was extracted using RNA iso plus reagent (TaKaRa). cDNA was prepared using TransScript II First-Strand cDNA Synthesis SuperMix kit (Transgen). Quantitative real-time PCR (qRT-PCR) analysis was performed using a TaqMan Probe Mix (Transgen) using a Bio-Rad IQ5 detection system. Primers used were listed in Table 1. Data showed mRNA levels relative to those of 18S, and normalized to the mean value of samples from control.

**Table 1:** Primers for RT-PCR

**Gene**	**Forward primer (5–3)**	**Reverse primer (5–3)**
Glut4	TCTCAATGGTTGGGAAGGAAA	GAACCGTCCAAGAATGAGTATCTC
MCK	CGGCTTCACTCTGGACGATG	TCTTATGCTTGTCTGTGGGTTTGT
myogenin	CCTGGAAGAAAAGGGACTGG	CGCTCAATGTACTGGATGGC
MHC1	CTCAAGCTGCTCAGCAATCTATTT	GGAGCGCAAGTTTGTCATAAGT
MHC2b	CAATCAGGAACCTTCGGAACAC	GTCCTGGCCTCTGAGAGCAT
FNDC5	CAGAAGAAGGATGTGCGGATGC	ACAGGCTCACTGGCTGGGCTCT
CTRP15	CCCCTTTATCCCATCTGAGGTTCTGC	GGCTACCCGAGGCTGGTGTAGTGAG
FGF21	GGAGATCAGGGAGGATGGAAC	TGGCTGTTGGCAAAGAAACC
IL6	GTTGCCTTCTTGGGACTGATG	CTGGCTTTGTCTTTCTTGTTATC
Atrogin1	CATCCCTGAGTGGCATCG	GAGTCTGGAGAAGTTCCCGTAT
MuRF1	ATCCTGCCCTGCCAACAC	CGGAAACGACCTCCAGACAT
18S	TTGACGGAAGGGCACCACCAG	GCACCACCACCCACGGAATCG

### Western blot

Cell lysates were subjected to SDS-PAGE and western blot analysis [23]. Briefly, cells were lysed with protein lysis buffer followed by heat denaturation. 20ug of whole cell proteins were applied to SDS-PAGE. After electrophoresis, the proteins were transferred to PVDF membranes, and blocked in the TBST buffer containing 5% nonfat dry milk for 1 hour at room temperature. The membranes were probed with the following different primary antibodies: anti-phosphorylated-Akt1 (Ser473) (Epitomics), anti-FNDC5 (Abcam), anti-phosphorylated-p70S6K (Ser371) (Cell signaling technology), anti-α-actin (ZSGB-BIO), anti-GAPDH (ZSGB-BIO) and anti-β-actin (ZSGB-BIO), and then washed and incubated with peroxidase-conjugated secondary antibody and finally visualized using Chemiluminescent HRP Substrate reagent (Millipore MA, USA) using an ECL detection system (Amersham Biosciences).

### Statistical analysis

Data, represented as the means ± SEM, were analyzed by the Student’s t-test for comparison of two groups or one-way ANOVA for multiple comparisons using the SPSS 17 software (SPSS Inc. Chicago, Illinois) to determine any significant differences. *p*<0.05 was considered significant.

## Results

### Palmitate induced insulin resistance in C2C12 myotubes

**Figure 1:**
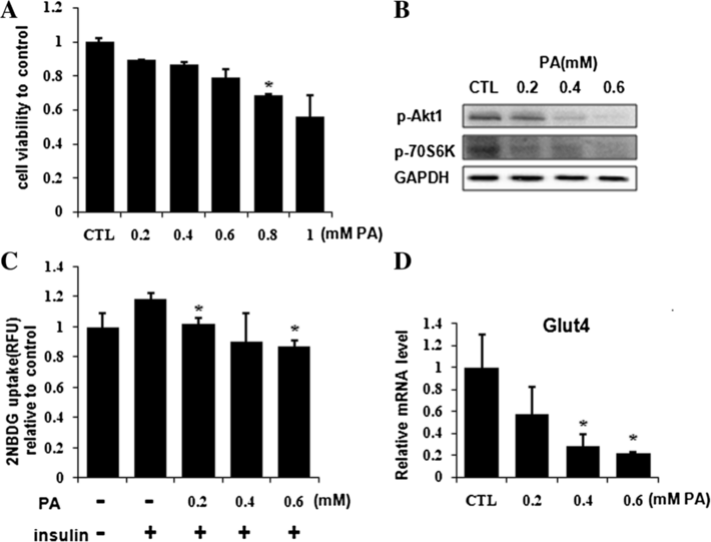
**Palmitate induced insulin resistance in C2C12 myotubes.** Myotubes were treated with indicated concentrations of palmitate for 24 hours. **(A)** Cell viability of C2C12 myotubes was evaluated by MTT assay. **(B)** Cells were stimulated with insulin (100 nM) for 15 min before harvest. Phosphorylated Akt1 (Ser473) and phosphorylated p70S6K (Ser371) were immunoprobed in western blot. GAPDH was detected as internal control. **(C)** 2NBDG uptake was carried out as described in Materials and Methods and intracellular fluorescence intensity was measured by a microplate reader. *p<0.05 *vs.* control stimulation with insulin. **(D)** The transcription of Glut4 gene was measured by qRT-PCR. The values were monitored by 18S and expressed as mean±SEM (n=3). *p<0.05 *vs.* control (CTL). PA, palmitate.

The inhibitory effect of chronic palmitate treatment on insulin/PI3K signaling pathway in myotubes was tested at first. The result of MTT assay showed that lower than 0.6 mM of palmitate did not significantly suppress the cell viability of C2C12 myotubes (Figure 1A). So, we chose 0.6 mM and lower concentrations of palmitate for next experiments. As shown, 0.2 to 0.6 mM of palmitate suppressed insulin-stimulated phosphorylation of Akt1 (Ser473) and p70S6K (Ser371) (Figure 1B). Correspondingly, palmitate inhibited insulin-stimulated 2NBDG uptake in a dose-dependent manner, i.e. 0.2 mM, 0.4 mM, 0.6 mM of palmitate inhibited 2NBDG uptake by 13.7%, 23.9%, 26.5%, respectively (Figure 1C). These concentrations of palmitate also decreased the transcription of glucose transporter 4 (Glut4) gene by 42%, 72%, 78%, respectively (Figure 1D). Taking together, our data suggest that 0.2 to 0.6 mM of palmitate reduce the insulin sensitivity of C2C12 myotubes.

### Palmitate, but not oleate, induced myotube loss in C2C12 myotubes

**Figure 2:**
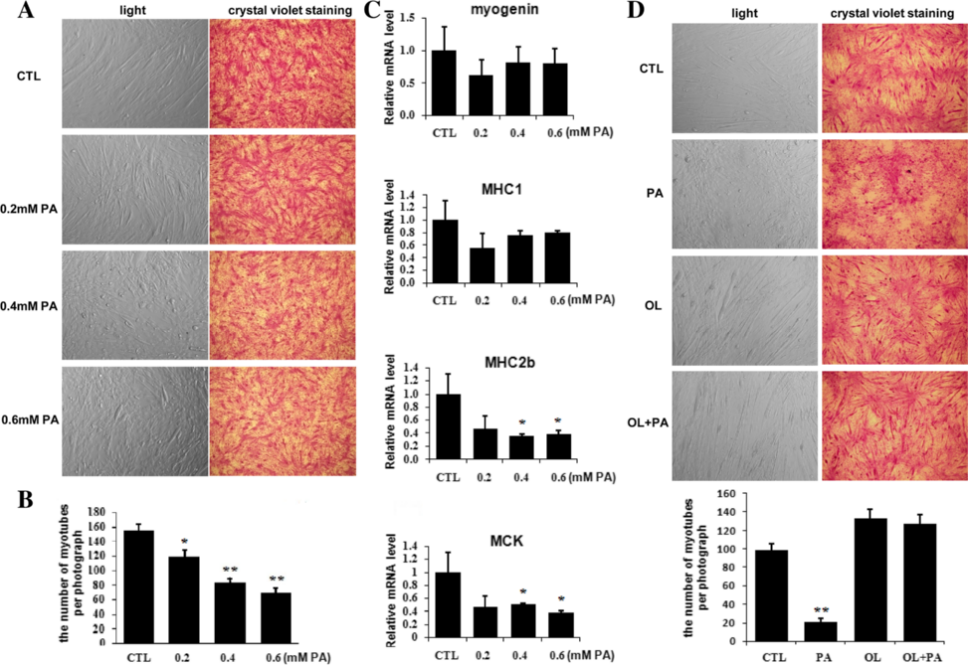
**Palmitate induced C2C12 myotube loss.** Myotubes were treated with palmitate or/and oleate for 24 hours. **(A)** Photographs were taken before (light) or after (crystal violet staining) 1% crystal violet staining under a phase contrast microscope; **(B)** The number of myotubes per photograph was counted after crystal violet staining (n=9). **(C)** The transcription of myogenin, MHC1, MHC2b and MCK genes was measured by qRT-PCR. The values were monitored by 18S and expressed as mean±SEM (n=3). **(D)** Photographs were taken like **(A)**, and the number of myotubes per photograph was counted. *p<0.05, **p<0.01 *vs.* CTL. PA, palmitate; OL, oleate; CTL, control.

Except insulin resistance, we noticed that palmitate had an apparent effect on morphous of myotubes (Figure 2A). We found that myocytes treated with 0.2 mM, 0.4 mM and 0.6 mM palmitate caused a significantly decrease in the number of myotubes by 14%, 41%, 49%, respectively (Figure 2B). In addition, the transcriptions of four marker genes relevant to muscle differentiation and myofiber composition, which are myogenin, MHC1, 2b and muscle creatine kinase (MCK), were suppressed by palmitate at different levels (Figure 2C). In the contrary, up to 0.6 mM concentrations of oleate, an unsatuated fatty acid, did not induce myotube loss, whenever it was used alone or together with palmitate (Figure 2D and unpresented data). These results demonstrate that palmitate induced myotube loss in C2C12 myotubes.

### Palmitate-induced myotube loss could not be duplicated by the blockage of PI3K pathway and p38 pathway

**Figure 3:**
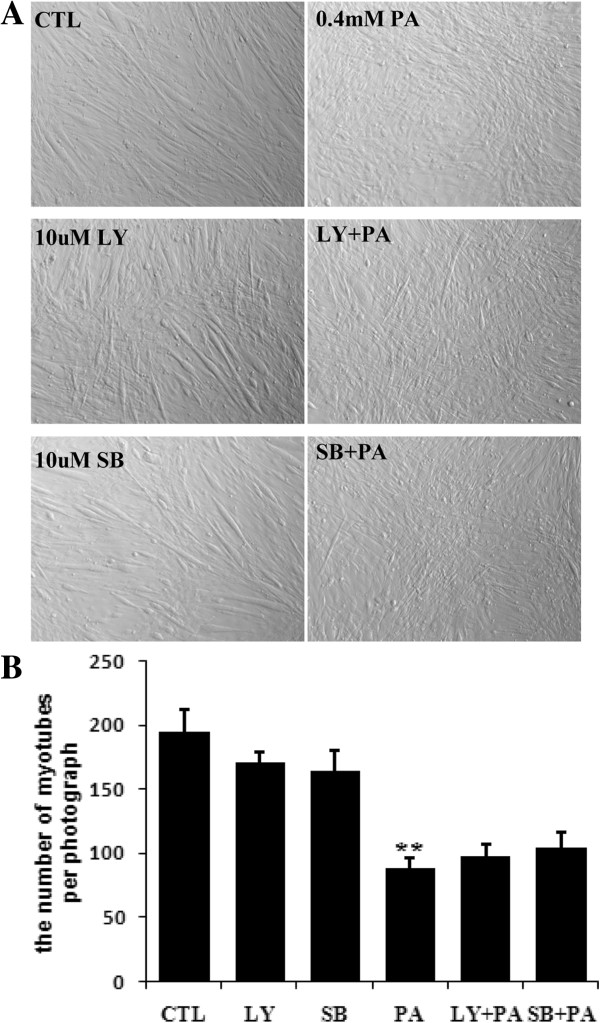
**Palmitate-induced myotube loss could not be duplicated by the blockage of PI3K and p38 pathways.** Myotubes were pretreated with 10 uM LY294002, or 10 uM SB203580 for 1 hour, followed by 0.4 mM palmitate for 24 hours. 0.1% DMSO as the control for inhibitor treatment, and BSA as the control for palmitate treatment. **(A)** Representative photographs taken under a phase contrast microscope; **(B)** The number of myotubes per photograph was counted after crystal violet staining. **p<0.01 *vs.* CTL. PA, palmitate; LY, LY294002; SB, SB203580.

PI3K- and p38-mediated pathways are known to participate in muscle differentiation and myotube fusion. So we presumed that blockage of these pathways may mimic palmitate-induced myotube loss. Unexpectedly, neither LY294002 (PI3K inhibitor) nor SB203580 (p38MAPK inhibitor) induced significant myotube loss in C2C12 myocytes like palmitate (Figure 3). These data demonstrate that the blockage of PI3K and p38 pathways by chemical inhibitors can not mimic the palmitate-induced myotube loss.

### Palmitate-induced myotube loss was associated with protein degradation

**Figure 4:**
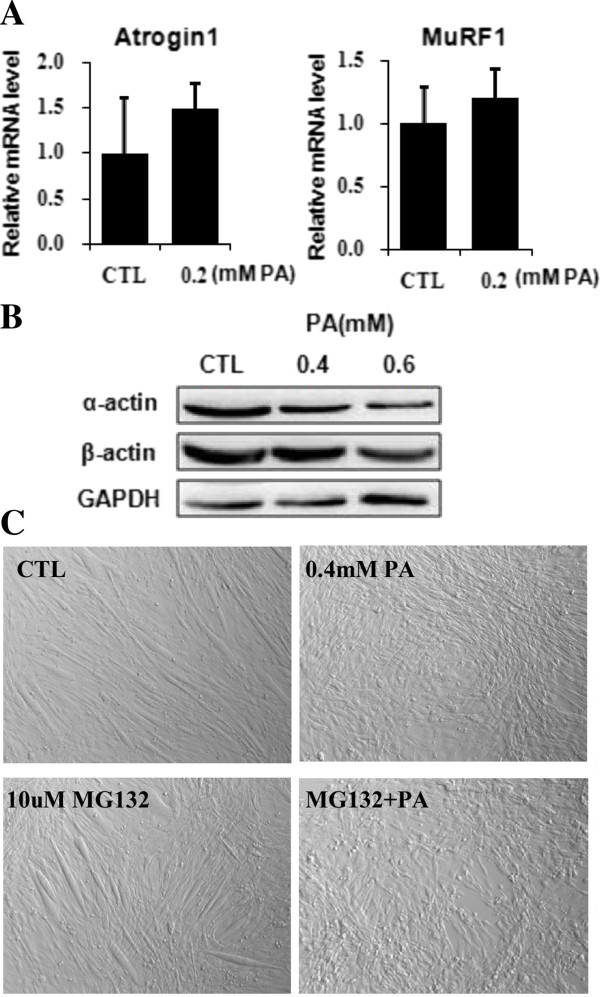
**Palmitate-induced myotube loss associated with protein degradation. (A)** Cells were treated with 0.2 mM palmitate for 24 hours, and BSA treatment was used as control. The transcription of Atrogin1 and MuRF1 genes was measured by qRT-PCR. **(B)** Cells were treated with palmitate for 24 hours and α-actin and β-actin protein levels were measured by western blot. GAPDH as control. **(C)** Cells were pretreated with 10 uM MG132 for 1 hour, followed by 0.4 mM palmitate treatment for 24 hours. DMSO and BSA treatments were used as controls for MG132 and palmitate, respectively. Representative photographs were shown. The values were expressed as mean±SEM (n=3).

To know whether palmitate-induced myotube loss was associated with increased proteolysis, we measured the transcription of two marker genes of proteasome-mediated protein degradation pathway, Atrogin1 and MuRF1 [24]. As shown, palmitate slightly increased the expression of Atrogin1 and MuRF1 genes (Figure 4A), but reduced the protein levels of α-actin and β-actin (Figure 4B). To know whether palmitate-induced myotube loss was proteasome-dependent, myotubes were pretreated with MG132 (proteasome inhibitor) prior to palmitate. As the results, 10 uM of MG132 for 1h did not prevent the myotube loss induced by palmitate, but showed apparent cytotoxicity and aggravated myotube loss (Figure 4C). Actually, we tested a wild range concentrations of MG132 for knowing its role in palmitate-induced myotube loss, In 1 uM to 5 uM of concentrations, MG132 was nontoxic but no impact on myotube morphology, either used alone or together with palmitate (data not shown); in 10 to 50 uM, MG132 was also nontoxic when used alone, but showed increasing toxicity with corresponding extents of cell death when used together with palmitate (data not shown). These results suggest that palmitate-induced myotube loss is associated with protein degradation, but the involvement of proteasome in this phenomenon need to be confirmed.

### Palmitate suppressed the expression of three health benefit myokine genes but promoted that of IL6 gene

**Figure 5:**
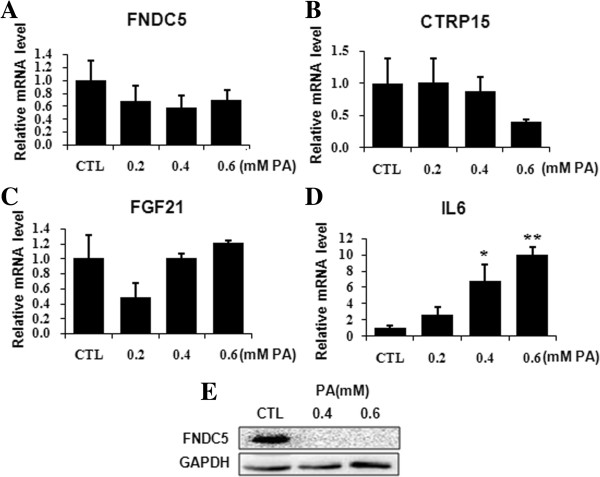
**Palmitate altered the expression of myokine genes in C2C12 myotbes.** Cells were treated with palmitate for 24 hours. The transcription of FNDC5 gene **(A)**, CTRP15 gene **(B)**, FGF21 gene **(C)** and IL6 gene **(D)** was measured by qRT-PCR and monitored via 18S. FNDC5 protein level was measured by western blot, GAPDH as control **(E)**. The values were expressed as mean±SEM (n=3). *p<0.05, **p<0.01 *vs.* CTL.

FNDC5, CTRP15 and FGF21 show health benefit roles in metabolism interference [18-20]. Up to now, the expression regulation about these myokines is largely unknown. To explore the connection between insulin resistance and the expression of these myokine genes, qRT-PCR assay was utilized. Palmitate suppressed the transcription of FNDC5 and CTRP15 genes (Figure 5A,B). However, palmitate showed a bidirectional influence to the transcription of FGF21 gene, being inhibitory at 0.2 mM concentration but stimulative at 0.4 mM and 0.6 mM concentration (Figure 5C). Oppositely, the expression of IL6 gene, encoding a pro-inflammatory cytokine which is also produced by muscle cells, was stimulated by palmitate in a dose-dependent manner (Figure 5D). We also detected the effect of palmitate on the expression of FNDC5 at protein level. As shown, 0.4 mM and 0.6 mM palmitate apparently reduced the protein level of FNDC5 (Figure 5E). Thus, palmitate impairs the expression of three health benefit myokine genes but promotes the expression of IL6 gene.

### The expression of myokine genes differently responded to pathway inhibitors

**Figure 6:**
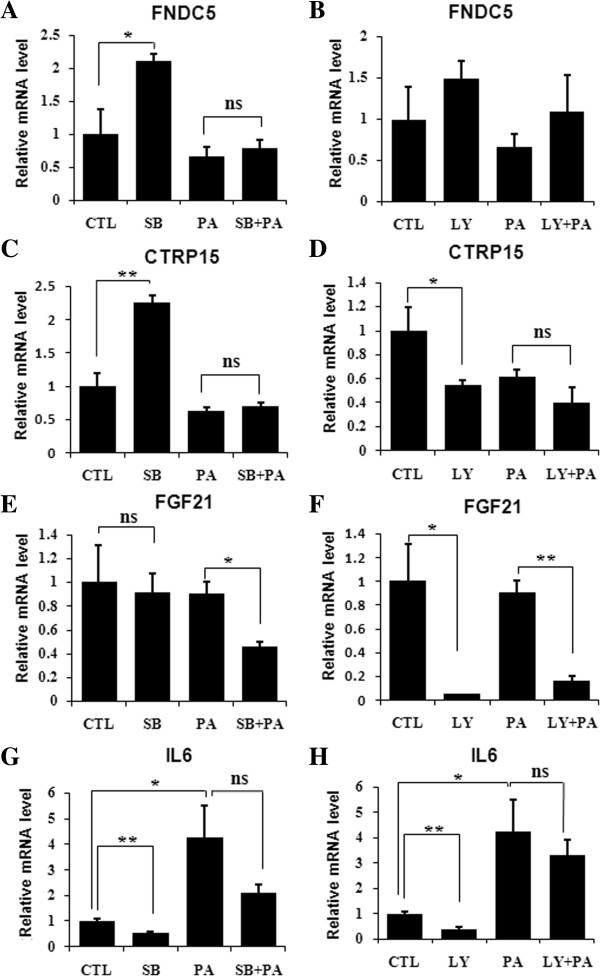
**The transcription of myokine genes differently responded to SB203580 and LY294002.** C2C12 myotubes were treated with 0.4 mM palmitate with or without chemical inhibitors for 24 hours. The transcription of FNDC5 gene **(A**, **B)**, CTRP15 gene **(C**, **D)**, FGF21 gene **(E**, **F)** and IL6 gene **(G**, **H)** was measured by qRT-PCR and monitored via 18S as internal control. The values were expressed as mean±SEM (n=3). *p<0.05, **p<0.01, ns, no significant. PA, 0.4 mM palmitate; SB, 10 uM SB203580; LY, 10 uM LY294002.

As an effort to know the potential mechanism underlying palmitate-altered expression of above myokines, chemical inhibitors of PI3K and p38 were used in the subsequent experiments. For FNDC5 gene, SB203580 significantly up-regulated the transcription of this gene in normal myotubes, but this up-regulation was blanked in the myotubes treated with palmitate (Figure 6A). Differently, LY294002 had no significant effect on the transcription of FNDC5 gene in both normal and palmitate-treated myotubes (Figure 6B). For CTRP15 gene, either SB203580 or LY294002 altered the transcription in normal not palmitate-treated myotubes, although the influence of SB203580 and LY294002 in normal myotubes was opposite (Figure 6C,D). For FGF21 gene, SB203580 did not affect the transcription in normal myotubes, but suppressed that in palmitate-treated myotubes; LY294002 exerted inhibitory effect in both normal and palmitate-treated myotubes (Figure 6E,F). Quite differently, the effect of SB203580 and LY294002 on the expression of IL6 gene was consistently negative in both normal and palmitate-treated myotubes (Figure 6G,H). These results demonstrate that the transcription of FNDC5, CTRP15, FGF21 genes differently respond to SB203580 and LY294002 in C2C12 myotubes.

## Discussion

Despite many studies have reported that palmitate can induce insulin resistance in myotubes, other phenotypes of palmitate in myotubes were not recognized well. In the present study, we explored the connection between palmitate treatment and two unwholesome phenomena, myotube loss and impaired expression of health benefit myokine genes, in C2C12 myotubes. Our results demonstrate for the first time that palmitate-induced insulin resistance in C2C12 myotubes is closely accompanied with myotube loss and the decreased expression of three health benefit myokine genes. These results is supportive for the notion that excessive concentration of saturated fatty acids in circulation is harmful for human healthy, although we know that palmitate treatment does not exactly fit the situation *in vivo*, where a mix of saturated and unsaturated fatty acids exists. For this reason, the role of other fatty acids existing in blood is necessary to be understand. Corresponding to the report that unsaturated fatty acid oleate can not stimulate insulin sensitivity like palmitate [25], our result showed that oleate is negative in the induction of myotube loss. This result is helpful to recognize the difference between saturated fatty acid and unsaturated fatty acid.

Skeletal muscle wasting often occurs with insulin resistance [26]. For example, Wang et al. found insulin resistance caused muscle wasting in db/db mice [26]. Randall et al. reported palmitate had negative effect on the myotube size and morphology of C2C12 myotubes [13]. As an effort to elucidate the connection between insulin resistance and myotube loss, we utilized C2C12 myotubes chronic exposed to palmitate as an insulin resistance model. To know the mechanism underlying palmitate-induced myotube loss, we evaluated the involvement of several signaling pathways in palmitate-induced myotube loss. Insulin/PI3K pathway is the first one, since previous report has shown that palmitate can suppress insulin-stimulated PI3K/Akt/mTOR pathway [9]. However, in our system, no evidence was obtained even a series of inhibitor-applied experiments were conducted, since three insulin/PI3K/mTOR pathway inhibitors, LY294002, wortmannin, rapamycin, did not result in myotube loss like palmitate (Figure 3 and nonpresented data) and on the other hand, two insulin/PI3K/mTOR pathway activators, PTEN inhibitor (VO-OHpic trihydrate) and mTOR activator (Phosphatidic acid), did not block palmitate-induced myotube loss (data not shown). We also concerned the involvement of PKC pathway, because one previous view is that palmitate can activate PKC in myotubes [9]. Unfortunately, we did not successfully set up the platform for PKC pathway inhibition experiment for practical reason. However, our finding about the different outcomes of palmitate and oleate on myotube loss may be a kind of indirect evidence supportive for the involvement of PKC in myotube loss, because it has shown that palmitate can be metabolized into DAG, a verified intracellular PKC activator, in myotubes, but diversely, oleate can only be metabolized to intracellular FFAs [27]. We understand that more direct evidence is needed to clear up the question. For example, PKC specific inhibitor- and PKC siRNA-involved strategise can be conducted. Actually, we have tried the use of Staurosporine as PKC inhibitor (data not shown). But later on, we realized that Staurosporine is not an efficient and specific PKC inhibitor. Meanwhile, we asked if p38 pathway connected to palmitate-induced myotube loss. The result is still negative. It is worth to note here that efficiencies of the chemical inhibitors and activators of PI3K and p38 pathways we used in this study have been confirmed, as they can obviously influence the differentiation of C2C12 myoblasts (data not shown).

Palmitate-induced myotube loss is certainly connected to protein degradation. The decline of protein level of α-actin and β-actin we found is a confident evidence since these two proteins are consistently expressed at transcriptional level but eliminated at protein level. As known, intracellular protein degradation are majorly attributed to two mechanisms: ubiquitin proteasome process (UPP) and lysosome-autophagy process [28]. Previous reports demonstrated that mytube loss and muscle wasting is related to UPP [26]. In present study, two lines of evidence are obtained. One is the decreased level of actin proteins, and the other is the increasing tendency of the expression of Atrogin1 and MuRF1genes, which encode two ubiquitin E3 ligases participating in UPP [24,26]. However, the experiment using proteasome inhibitor MG132 needs to be further optimized, as we do not know at this moment why the MG132 concentration necessary for proteasome inactivation (10–50 uM) were toxic when combined with palmitate. Similar to previous reports, up to 50 uM of MG132 was safe for the cells when added alone [29]. For this cytotoxicity of MG132 occurring in palmitate-treated cells, one interpretation is considerable, as it’s reported that strong proteasome malfunction can induce severe autophagic cell death [30]. So, it is promising to detect whether palmitate-induced myotube loss is closely related to autophagy process.

Another attempt of this study is to explore the role of palmitate in the production of several myokines. Even preliminary, this attempt is important for enriching our knowledge about myokine production particularly about the influence of insulin resistance in myokine production, which is actually a barren land up to now. Our finding is interesting, because it shows for the first time that palmitate-induced insulin resistant accompany with impaired expression of three healthy benefit-oriented myokines. These three myokines are: (1) Irisin, a secretory portion of FNDC5 protein, being able to positively promote the browning of white adipose tissue and improve insulin sensitivity in both human and mice [19]; (2) CTRP15, also known as myonectin, being able to mediate the cross-talk between skeletal muscle and other metabolic compartments, such as adipose tissue and liver, to coordinate the integration of whole-body metabolism [18]; (3) FGF21, a known endogenous regulator for systemic glucose and lipid metabolism [20]. In our study, palmitate-inhibited the expression of FNDC5 gene was evidenced at both mRNA and protein levels, while the suppressive effect of palmitate on the expression of CTRP15 and FGF21 genes was observed only at mRNA level because of no available antibodies. From previous studies, a few coincidant clues can be found. For example, high fat diet inhibited the expression of CTRP15 gene in mice [18].

The signal pathways related to palmitate-suppressed expression of myokine genes were briefly studied. The results were informative but not conclusive. One valuable point, from our view at least, is the elimination of the effect of SB203580 and LY294002 by palmitate. It is that p38 inhibitor SB203580 up-regulated the transcription of FNDC5 and CTRP15 genes in normal myotubes but not in palmitate-treated myotubes. We consider that two pieces of information can be extracted from these results: the first, p38 inactivation is important for the expression of these two genes; the second, palmitate-suppressed expression of these two genes seems corelated to its role in p38 activation. In fact, palmitate-induced p38 activation has got reported by others [31]. Anyway, we got from this study that the three checked myokine genes have their own response patterns upon pathway inhibitors, implying the regulation mechanisms of these genes are different. As to the transcription of FNDC5 and CTRP15 genes, p38 pathway is predominantly involved; for the transcription of FGF21 gene expression, however, PI3K pathway is apparent relevant.

## Conclusions

In summary, palmitate-induced insulin resistance is associated with myotube loss and impaired expression of three health benefit myokine genes (FNDC5, CTRP15 and FGF21) in C2C12 myotubes. These findings provide new evidence for the negative impact of high concentration palmitate in muscle cells. Further studies are needed to investigate the underlying mechanism.

## Abbreviations

PA: Palmitate; PFA: Paraformaldehyde; FFA: Free fatty acids; BSA: Bovine serum albumin; PKC: Protein kinase C; IRS-1: Insulin receptor substrate-1; Glut4: Glucose transporter 4; MHC: Myosin heavy chain; MCK: Muscle creatine kinase; FGF21: Fibroblast growth factor-21; UPP: Ubiquitin proteasome process.

## Competing interests

The authors declare that they have no competing interests.

## Authors’ contributions

HX and MY conceived and designed the study. MY, DW and XW carried out the experiments and analyzed the data. HX helped conducting the experiments. HX and MY wrote the manuscript. All authors read and approved the manuscript.

## References

[B1] DeFronzo RA Ferrannini E Insulin resistance. A multifaceted syndrome responsible for NIDDM, obesity, hypertension, dyslipidemia, and atherosclerotic cardiovascular disease Diabetes Care 1991 14 3 173 94 10.2337/diacare.14.3.173 2044434

[B2] Jove M Palmitate-induced interleukin 6 production is mediated by protein kinase C and nuclear-factor kappaB activation and leads to glucose transporter 4 down-regulation in skeletal muscle cells Endocrinology 2005 146 7 3087 95 10.1210/en.2004-1560 15802498

[B3] Boden G Role of fatty acids in the pathogenesis of insulin resistance and NIDDM Diabetes 1997 46 1 3 10 10.2337/diabetes.46.1.3 8971073

[B4] Roden M Mechanism of free fatty acid-induced insulin resistance in humans J Clin Invest 1996 97 12 2859 65 10.1172/JCI118742 8675698 PMC507380

[B5] Dimopoulos N Differential effects of palmitate and palmitoleate on insulin action and glucose utilization in rat L6 skeletal muscle cells Biochem J 2006 399 3 473 81 10.1042/BJ20060244 16822230 PMC1615906

[B6] Holland WL Inhibition of ceramide synthesis ameliorates glucocorticoid-, saturated-fat-, and obesity-induced insulin resistance Cell Metab 2007 5 3 167 79 10.1016/j.cmet.2007.01.002 17339025

[B7] Dey D Involvement of novel PKC isoforms in FFA induced defects in insulin signaling Mol Cell Endocrinol 2006 246 1–2 60 4 16448741 10.1016/j.mce.2005.12.014

[B8] Yu C Mechanism by which fatty acids inhibit insulin activation of insulin receptor substrate-1 (IRS-1)-associated phosphatidylinositol 3-kinase activity in muscle J Biol Chem 2002 277 52 50230 6 10.1074/jbc.M200958200 12006582

[B9] Deng YT Suppression of free fatty acid-induced insulin resistance by phytopolyphenols in C2C12 mouse skeletal muscle cells J Agric Food Chem 2012 60 4 1059 66 10.1021/jf204496f 22191431

[B10] Long YC Zierath JR AMP-activated protein kinase signaling in metabolic regulation J Clin Invest 2006 116 7 1776 83 10.1172/JCI29044 16823475 PMC1483147

[B11] Brown DM Parr T Brameld JM Myosin heavy chain mRNA isoforms are expressed in two distinct cohorts during C2C12 myogenesis J Muscle Res Cell Motil 2012 32 6 383 90 10.1007/s10974-011-9267-4 22012579

[B12] Vescovo G Skeletal muscle myosin heavy chain expression in rats with monocrotaline-induced cardiac hypertrophy and failure. Relation to blood flow and degree of muscle atrophy Cardiovasc Res 1998 39 1 233 41 10.1016/S0008-6363(98)00041-8 9764203

[B13] Randall WB Docosahexaenoic acid protects muscle cells from palmitate-induced atrophy 2012 ISRN Obesity 10.5402/2012/647348 PMC3914282 24533207

[B14] Fanzani A Molecular and cellular mechanisms of skeletal muscle atrophy: an update J Cachexia Sarcopenia Muscle 2012 3 3 163 79 10.1007/s13539-012-0074-6 22673968 PMC3424188

[B15] Goldbach-Mansky R Immunology in clinic review series; focus on autoinflammatory diseases: update on monogenic autoinflammatory diseases: the role of interleukin (IL)-1 and an emerging role for cytokines beyond IL-1 Clin Exp Immunol 2012 167 3 391 404 10.1111/j.1365-2249.2011.04533.x 22288582 PMC3374271

[B16] Zhang L Pharmacological inhibition of myostatin suppresses systemic inflammation and muscle atrophy in mice with chronic kidney disease FASEB J 2011 25 5 1653 63 10.1096/fj.10-176917 21282204 PMC3079306

[B17] Pedersen BK The diseasome of physical inactivity–and the role of myokines in muscle–fat cross talk J Physiol 2009 587 Pt 23 5559 68 19752112 10.1113/jphysiol.2009.179515 PMC2805368

[B18] Seldin MM Myonectin (CTRP15), a novel myokine that links skeletal muscle to systemic lipid homeostasis J Biol Chem 2012 287 15 11968 80 10.1074/jbc.M111.336834 22351773 PMC3320944

[B19] Bostrom P A PGC1-alpha-dependent myokine that drives brown-fat-like development of white fat and thermogenesis Nature 2012 481 7382 463 8 10.1038/nature10777 22237023 PMC3522098

[B20] Kim KH Autophagy deficiency leads to protection from obesity and insulin resistance by inducing Fgf21 as a mitokine Nat Med 2013 19 1 83 92 23202295 10.1038/nm.3014

[B21] Cheng Q Visfatin inhibits apoptosis of pancreatic beta-cell line, MIN6, via the mitogen-activated protein kinase/phosphoinositide 3-kinase pathway J Mol Endocrinol 2011 47 1 13 21 10.1530/JME-10-0106 21471274

[B22] Jove M Palmitate induces tumor necrosis factor-alpha expression in C2C12 skeletal muscle cells by a mechanism involving protein kinase C and nuclear factor-kappaB activation Endocrinology 2006 147 1 552 61 16223857 10.1210/en.2005-0440

[B23] Li N D-galactose induces necroptotic cell death in neuroblastoma cell lines J Cell Biochem 2011 112 12 3834 44 10.1002/jcb.23314 21826710

[B24] Zhou Q Evidence for adipose-muscle cross talk: opposing regulation of muscle proteolysis by adiponectin and Fatty acids Endocrinology 2007 148 12 5696 705 10.1210/en.2007-0183 17761767

[B25] Chavez JA Summers SA Characterizing the effects of saturated fatty acids on insulin signaling and ceramide and diacylglycerol accumulation in 3T3-L1 adipocytes and C2C12 myotubes Arch Biochem Biophys 2003 419 2 101 9 10.1016/j.abb.2003.08.020 14592453

[B26] Wang X Insulin resistance accelerates muscle protein degradation: activation of the ubiquitin-proteasome pathway by defects in muscle cell signaling Endocrinology 2006 147 9 4160 8 10.1210/en.2006-0251 16777975

[B27] Gaster M Rustan AC Beck-Nielsen H Differential utilization of saturated palmitate and unsaturated oleate: evidence from cultured myotubes Diabetes 2005 54 3 648 56 10.2337/diabetes.54.3.648 15734839

[B28] Lilienbaum A Relationship between the proteasomal system and autophagy Int J Biochem Mol Biol 2013 4 1 1 26 23638318 PMC3627065

[B29] Sadiq F Amino acids and insulin act additively to regulate components of the ubiquitin-proteasome pathway in C2C12myotubes BMC Mol Biol 2007 8 23 10.1186/1471-2199-8-23 17371596 PMC1845170

[B30] Sheng X Different degree in proteasome malfunction has various effects on root growth possibly through preventing cell division and promoting autophagic vacuolization PLoS One 2012 7 9 e45673 10.1371/journal.pone.0045673 23029176 PMC3448697

[B31] Kadotani A Different impacts of saturated and unsaturated free fatty acids on COX-2 expression in C(2)C(12) myotubes Am J Physiol Endocrinol Metab 2009 297 6 E1291 303 10.1152/ajpendo.00293.2009 19755671

